# Acute Twin-to-Twin Transfusion Syndrome Resulting in Fetal Bradycardia and Neonatal Death: A Case Report

**DOI:** 10.3390/medicina58121813

**Published:** 2022-12-09

**Authors:** Mi Ju Kim, Hyun Mi Kim, Hyun-Hwa Cha, Haemin Kim, Hyo-Shin Kim, Won Joon Seong

**Affiliations:** 1 Department of Obstetrics and Gynecology, Kyungpook National University Hospital, School of Medicine, Kyungpook National University, Daegu 41944, Republic of Korea; 2 Department of Obstetrics and Gynecology, Kyungpook National University Chilgok Hospital, School of Medicine, Kyungpook National University, Daegu 41404, Republic of Korea

**Keywords:** acute twin-to-twin transfusion syndrome, anemia of donor twin, fetal bradycardia, monochorionic twin, neonatal death

## Abstract

In monochorionic twins with no evidence of chronic twin-to-twin transfusion syndrome or twin anemia-polycythemia sequence, a sudden onset of fetal transfusion syndrome after the second trimester of pregnancy is defined as acute twin-to-twin transfusion syndrome. Labor pain, change in the fetal position, and birth order are known risk factors for this condition, and the hemoglobin level of the donor twin is usually reported to be <12 g/dL. We report a recent case of acute twin-to-twin transfusion syndrome without effective labor pain causing cervical changes, resulting in fetal bradycardia and neonatal death after birth; however, the anemia of the donor twin was not as severe as has been reported previously in twin-to-twin transfusion syndrome cases.

## 1. Introduction

The incidence of monochorionic twins is lower than that of dichorionic twins in twin pregnancies. Monochorionic twins are, however, more likely to be associated with obstetric and neonatal complications [1,2]. Almost all cases of monochorionic twins have a placental vascular connection between the two fetuses, resulting in either chronic twin-to-twin transfusion syndrome (TTTS) or a spontaneous twin anemia-polycythemia sequence (TAPS) [3,4]. In very rare cases of monochorionic twins, where there is no evidence of chronic TTTS or TAPS, a sudden onset of fetal transfusion syndrome after the second trimester of pregnancy is defined as acute TTTS or acute peripartum TTTS [3,5,6].

Here we report on a recent case of acute TTTS occurring without evident risk factors and resulting in very poor outcomes after birth.

## 2. Case Report

**Figure 1:**
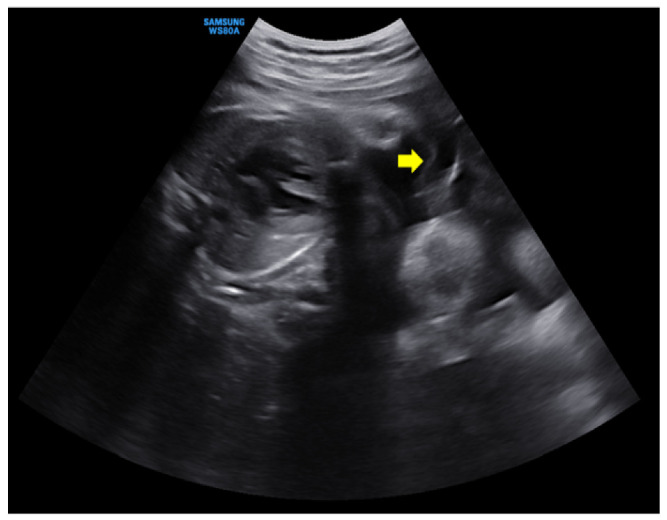
An ultrasonographic examination at 6 days before visiting emergency room. The intertwine membrane (yellow arrow) was well seen, and the amniotic fluid of both fetuses was normal.

A 28-year-old pregnant woman (gravida 1, para 0), who had attended regular prenatal checkups at our hospital outpatient clinic, visited our emergency room at 35 + 1 weeks of gestation because of persistent uterine contractions for 3 h. She felt fetal movements relatively distinctly and experienced no other obstetric problems such as vaginal bleeding or a vaginal watery discharge. She reported that she had felt her abdomen suddenly enlarge two days before, and that from then on, her dyspnea seemed to worsen. Her medical history was unremarkable, and her pregnancy had involved a spontaneous monochorionic diamniotic twin gestation. Amniocentesis had been performed because of the high risk of Down syndrome in the integrated test, but the patient had been diagnosed with a normal karyotype, and there were no structural abnormalities on detailed ultrasonography at 20 weeks of gestation. Ultrasonographic findings showed no marked differences in weight between the two fetuses and no significant differences in the amniotic fluid volume; therefore, the possibility of chronic TTTS was considered low. However, a short cervix was confirmed and a progesterone vaginal tablet was used. Growth delays were observed in both fetuses from 29 weeks of gestation, but there were no specific findings in infectious serum markers for congenital infections, and no significant problems in the umbilical artery, middle cerebral artery doppler, and amniotic fluid volume were observed. Serial ultrasonography was consequently performed every week. And, an ultrasound examination performed at an outpatient clinic 6 days before visiting the hospital showed an intertwin membrane, and the amniotic fluid volume were normal in both fetuses (Figure 1).

**Figure 2:**
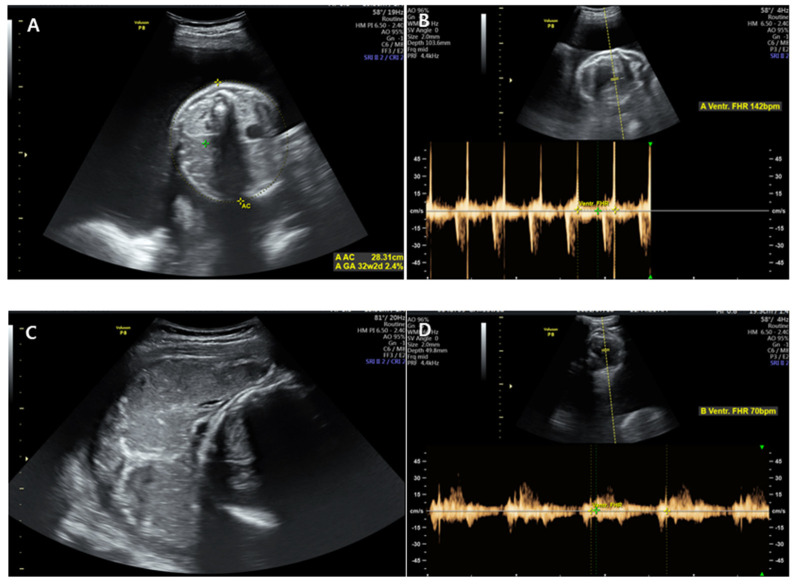
Ultrasonography was performed at admission. (**A**,**B**) showed the first fetus located on the left side of the mother. The fetal heart rate was 140–160 beats/min with good fetal motion, and amniotic fluid volume index (AFI) was more than 30 cm, showing polyhydramnios. (**C**,**D**) showed the second fetus located on the right side of the mother with breech presentation. The heart rate was 70–80 beats/min, showing a consistent bradycardia.

Initially, the patient’s vital signs were stable and as follows: blood pressure, 125/75 mmHg; pulse rate, 98 beats/min; respiratory rate, 20 breaths/min; body temperature, 36.6 °C; and oxygen saturation, 99%. Cervical examination revealed 10% cervical dilatation, 50% effacement, and −2 fetal station. Ultrasonography showed that the first fetus was located on the left side of the mother in a vertex presentation and that the fetal heart rate was 150–160 beats/min with good fetal motion, but the amniotic fluid volume index (AFI) was more than 30 cm, showing polyhydramnios. The second fetus was located on the right side of the mother and exhibited a heart rate of 70–80 beats/min and consistent bradycardia with a breech presentation, showing oligohydramnios (Figure 2). No evidence of placental abruption was noted. Continuous fetal monitoring was performed while preparing for an emergency cesarean section, and continuous fetal bradycardia of the second fetus was noted as 30–40 mmHg of uterine contractions at 5–6-min intervals.

An emergency cesarean section was performed under general anesthesia. The first neonate was delivered with a weight of 1820 g, APGAR scores of 5 and 7 at 1 and 5 min, respectively, and an umbilical arterial pH of 7.331. The second neonate weighed 1510 g and had APGAR scores of 1 and 1 at 1 and 5 min, respectively, and an umbilical arterial pH of 7.164 with anemic features. The placenta weighed 1180 g. No specific findings such as abruption were observed visually, and no specific findings on the placenta and umbilical cord, other than a superficial anastomosis were confirmed on pathological biopsy.

The initial laboratory test of the first neonate revealed a hemoglobin (Hb) level of 23.6 g/dL, hematocrit (Hct) of 67.7%, and reticulocyte count of 5.27%. The infant was discharged after 26 days without specific complications after ventilator care and phototherapy, and at 8 months of age at the time of writing, is currently exhibiting good growth and development. The second infant showed an initial Hb level of 15.2 g/dL, Hct of 47.1%, and a reticulocyte count of 5.66%, and received intensive care due to severe acidosis and hypoxic ischemic encephalopathy, but died 3 days after birth. The mother was discharged from the hospital 4 days after delivery without any complications.

## 3. Discussion

Monochorionic twins are more likely to have obstetric and neonatal complications than dichorionic twins [1.2]. In monochorionic twins, two fetuses share a single placenta. This may lead to growth discordance because the two fetuses receive blood supply from different areas of the placenta [7]. In addition, in almost all monochorionic twin cases, there exists a placental vascular connection between the two fetuses, which results in either chronic TTTS or spontaneous TAPS [3,4]. Chronic TTTS occurs in approximately 5–9% of monochorionic twins and is usually diagnosed in the second trimester of pregnancy [2]. Oligohydramnios occur in the donor twin, and polyhydramnios occur in the recipient twin by vascular anastomosis. In some cases, fetal therapy such as laser ablation can be performed with good results [2]. TAPS refers to the occurrence of anemia-polycythemia in monochorionic twins, without evidence of the oligo-polyhydramnios sequence, which occurs after incomplete laser ablation or natural course, usually with a reticulocyte count ratio of ≥1.7. It is diagnosed using the middle cerebral artery peak systolic velocity of the two fetuses [4,8].

The diagnostic criteria for acute TTTS are a difference in Hb level of ≥8 g/dL between fetuses at birth, and a normal reticulocyte count in the neonates [9]. In our case, six days before the acute TTTS occurred, the AFI of both fetuses in the ultrasound was normal, so it is not considered to be a chronic TTTS. During the event, the two fetuses showed a polyhydromnios-oligohydromanios sequence, and the Hb differences between the two fetus was confirmed to be 8.3 g/dL, which can be considered to meet the diagnostic criteria of acute TTTTS.

In some studies, only cases of delivery after 31 weeks of gestation were defined as having acute peripartum TTTS [6]. Acute peripartum TTTS occurs in 1.5–2.5% of monochorionic twins [3,9] and may occur as a result of changes in blood pressure caused by labor pain and fetal position change, resulting in rapid blood transfer between the fetuses [10,11]. In previous studies, most cases of acute TTTS occurred after vaginal delivery, and according to a report by Lopriore et al., 100% of acute TTTS cases were delivered through vaginal delivery [3]. However, in our case, labor pain lasted for a relatively short period and was not strong enough to cause cervical changes, but unlike previous reports, it resulted in rapid anemia and negative neonatal outcome in the donor fetus. In addition, considering that the mother had a sudden increase in her abdomen size and dyspnea two days before the visit, the possibility of acute TTTS occurring before labor pain cannot be ruled out. Suzuki reported acute TTTS after an elective cesarean section without labor, which he explained was due to hypotension and a change in intrauterine pressure caused by the spinal anesthesia, resulting in a blood pressure difference between the two fetuses [9]. In this case, delivery was performed with general anesthesia, but since fetal anemia was already suspected before anesthesia, it is conjectured that the anesthesia may have worsened the condition, although it is uncertain whether it was the cause of the acute TTTS. Several studies have reported that birth order also affects the occurrence of acute TTTS, and in most cases, the donor twin is the first infant [3], however, in this report, the donor twin was the second infant. Therefore, it is considered that the occurrence of acute TTTS may have other causes in addition to differences in pressure caused by labor pain and birth order.

Previous studies have reported an Hb level of <12.0 g/dL in the anemic fetus in acute TTTS, but in this case, although the Hb level was 15 g/dL, which is higher than in other cases, the prognosis remained poor. There may be several reasons for this. First, low birth weight and growth restrictions on the affected fetus may have caused severe fetal hypoxia, even though the anemia was not severe. Growth restriction is a risk factor for fetal death in twins [2], and infants with growth restriction may lack the ability to withstand even small external insults [2]. Second, it seems that there was not enough time for the Hb level to reveal distress as serious anemia emerged very rapidly. Finally, when the umbilical cord of the first fetus was clamped during delivery, the blood remaining in the umbilical cord flowed back to the placenta. If a uterine contraction occurred at this time, the blood would have entered the umbilical cord of the second fetus. The umbilical cord of the second fetus manifested a relatively lower pressure than the placenta, through vascular anastomosis, thus exhibiting a higher Hb level than the actual value.

## 4. Conclusions

It should be noted that even if there is no evidence of chronic TTTS or TAPS in monochorionic twins, acute progressive TTTS may occur. Therefore, when treating monochorionic twins, it should be kept in mind that even weekly prenatal checkups cannot completely prevent morbidities caused by acute TTTS. It is important to keep in mind that the prognosis of acute-onset TTTS may be even worse, especially if there is fetal growth restriction. In addition, further research is needed to predict acute TTTS in advance in monochorionic twins.

## Data Availability

The datasets used and/or analyzed during the current study are available from the corresponding author on reasonable request.
